# Complete response with combined BRAF and MEK inhibition in BRAF mutated advanced low-grade serous ovarian carcinoma

**DOI:** 10.1080/03009734.2020.1826612

**Published:** 2020-10-10

**Authors:** Bengt Tholander, Anthoula Koliadi, Johan Botling, Hanna Dahlstrand, Anne Von Heideman, Håkan Ahlström, Kjell Öberg, Gustav J. Ullenhag

**Affiliations:** a Department of Oncology, Uppsala University Hospital, Uppsala, Sweden; b Department of Immunology, Genetics and Pathology, Science for Life Laboratory, Uppsala University, Uppsala, Sweden; c Department of Surgical Sciences, Uppsala Sweden, Division of Radiology, Uppsala University Hospital, Uppsala, Sweden; d Department of Oncologic Endocrinology, Uppsala University Hospital, Uppsala, Sweden; e Department of Medical Sciences, Uppsala University, Uppsala, Sweden

**Keywords:** BRAF inhibitor, chemotherapy, low-grade serous ovarian cancer, MEK inhibitor, next-generation sequencing, surgery, targeted therapy, V600E mutation

## Abstract

More effective treatments are needed for low-grade serous ovarian carcinoma (LGSOC). Our patient, who suffers from metastatic LGSOC, had received all established treatments. Sequencing analysis revealed an activating BRAF mutation. Therefore, combined treatment with BRAF and MEK inhibitors, which is the gold standard in malignant melanoma, was initiated. After eight months of therapy, the response was assessed as complete and the treatment is still, 3.5 years after initiation, of benefit. To our knowledge, no complete response on combined BRAF and MEK inhibitor treatment of low-grade serous ovarian cancer has previously been reported.

## Introduction

Low-grade serous ovarian carcinoma (LGSOC) is a less common subtype, affecting around 5% of all patients with epithelial ovarian cancer (1). However, in contrast to high-grade serous ovarian carcinoma (HGSOC), LGSOC more often affects young, fertile women. Low-grade serous ovarian carcinoma has also different clinical and molecular biologic characteristics and course of disease, compared to HGSOC. Primary surgery is standard of care, and is often curative in LGSOC, but both early and late relapses are common. In stages IC–IV, postoperative platinum-based chemotherapy is recommended, but the response is often poor, especially at relapse (2). Repeated surgeries with curative or palliative intent are regularly needed. However, the course of disease may be indolent, with slow progression over many years, and with periods with stable disease even without treatment. Hormonal therapy may be effective (3). Nevertheless, therapy resistance, disease generalization, and progression often finally lead to death. There is need for more effective treatments for patients with LGSOC, possibly individually tailored, molecular biology-driven targeted therapies (4).

Mutations in BRAF, KRAS, and NRAS genes have been detected in LGSOC (4). In serous borderline tumours, the pre-malignant form preceding LGSOC, BRAF mutations are present in almost half of the cases (5). In HGSOC, BRAF mutations are rare (6), while the reported frequency of BRAF mutations is 5–14% in LGSOC (5,7). Treatment with a MEK inhibitor alone has been compared to chemotherapy in a clinical randomized trial in LGSOC patients regardless of BRAF status. A median progression-free survival (PFS) of 13 months, compared to 7 months for the group receiving physician’s choice, was achieved (8). In a phase 3 study (MEK inhibitor in low-grade serous ovarian cancer, MILO), patients with LGSOC were randomized to treatment with the MEK inhibitor binimetinib or chemotherapy. There were no significant differences for the primary endpoint PFS (9). A few cases with responses on BRAF inhibitor treatment have been reported in ovarian cancer (7). Furthermore, long-term survival has been reported in a patient with LGSOC treated with the BRAF inhibitor vemurafenib (10). In addition, there are a few recent reports on BRAF and MEK inhibitor combination therapy in patients with LGSOC (6,11).

Combination treatment with BRAF and MEK inhibitors is recommended in patients with BRAF mutated malignant melanoma. Combining dabrafenib and the MEK inhibitor trametinib or the BRAF inhibitor encorafinib with the MEK inhibitor binimetinib results in impressive and durable responses and also prolonged survival in patients with metastatic melanoma (12–14).

One might assume that all patients with BRAF mutant cancer would benefit from treatment with BRAF inhibitors. However, colon cancer patients harbouring the BRAF oncogenic lesion have a poor prognosis and do not respond to vemurafenib therapy. It was shown that this resistance is mediated through feedback activation of EGFR (15).

## Case presentation

### Patient characteristics, course of disease, and conventional treatments received

Our patient was diagnosed in 1986, at the age of 20, with serous borderline tumour, FIGO (International Federation of Gynaecology and Obstetrics) stage IC at Uppsala University Hospital. She had bilateral ovarian masses, both 8 cm in diameter with no capsules, but rough, rugged surface. Furthermore, loose tissue in the pouch of Douglas and 5 litres of ascites were found. Macroscopically radical primary surgery was performed, with bilateral salpingo-oophorectomy, hysterectomy, and omentectomy. No microscopic invasion in the ovaries, neither in the pelvic peritoneum nor the omentum, and no macro- or microscopic carcinomatosis were found. Postoperative chemotherapy with seven cycles of doxorubicin and cisplatin was delivered. Second-look surgery revealed no residual disease, and no further therapy was given. The patient was prescribed oral oestrogen and had regular follow-ups during 11 years with clinical examinations and serum levels of the tumour marker CA-125, without signs of recurrence. However, in 1997, first relapse was evident in lymph nodes in the abdomen and the left groin, with node calcifications on CT scan. The nodes in the groin were removed. Relapse was verified, but now with invasive LGSOC. The patient was treated with seven cycles of paclitaxel and carboplatin. The disease was stable at evaluation. New nodal progression was evident in 1999, which prompted hormonal therapy with tamoxifen and medroxyprogesterone, and this treatment turned out effective. In 2003, lymph node progression was again verified, now also in the left axillary and supraclavicular nodes. Treatment with uracil and tegafur resulted in a durable partial response, lasting to 2010. Thereafter, a slow continuous and symptomatic nodal progression was evident, prompting repeated palliative abdominal surgeries. Neither weekly paclitaxel plus bevacizumab treatment nor MTOR inhibition with everolimus was effective.

### Initiation, conduct, and modulation of combined BRAF and MEK inhibitor therapy

**Figure 1.**
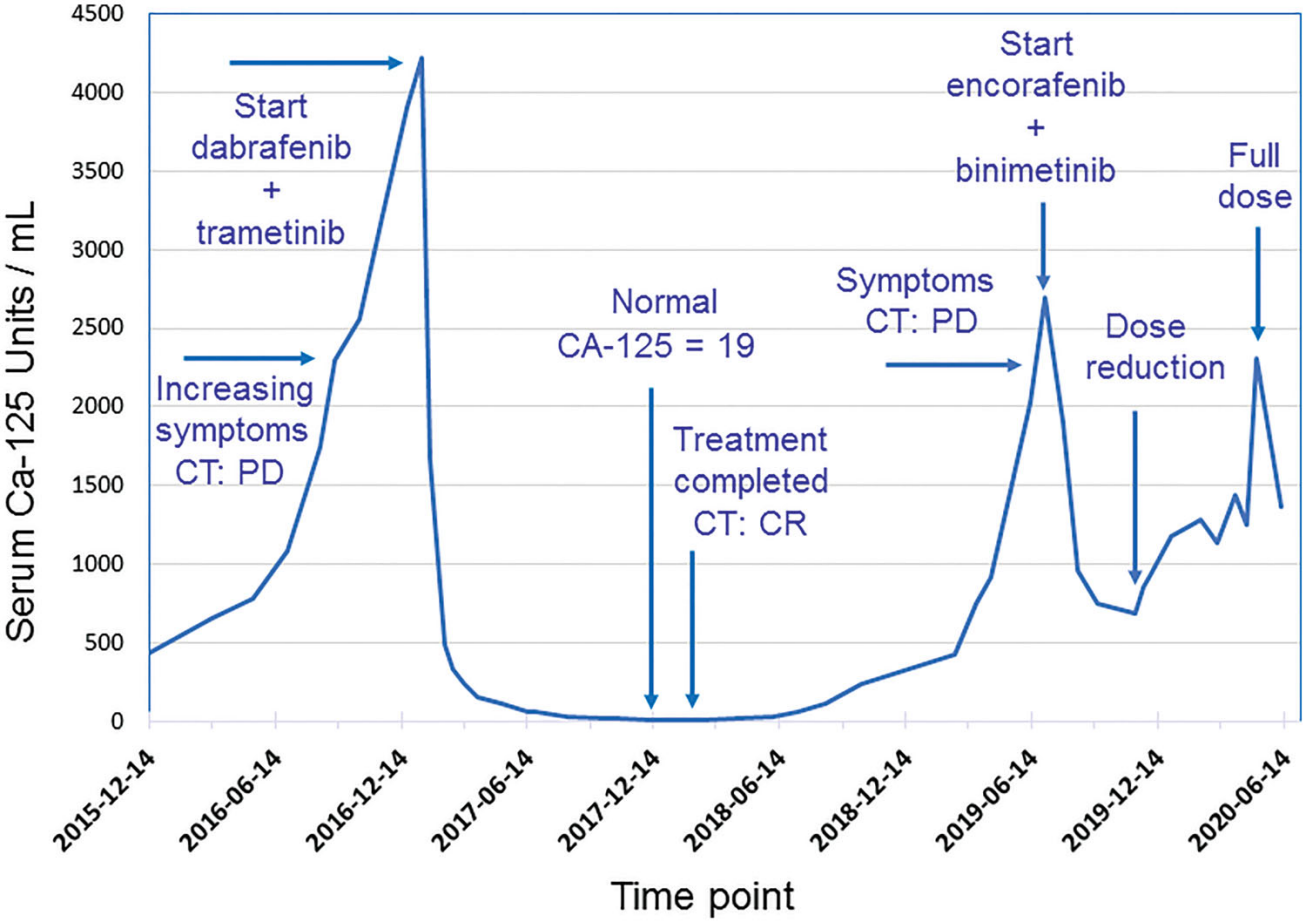
Serum levels of CA-125 during course of treatment with combination of BRAF and MEK inhibitors in a patient with low-grade serous ovarian cancer.

**Figure 2.**
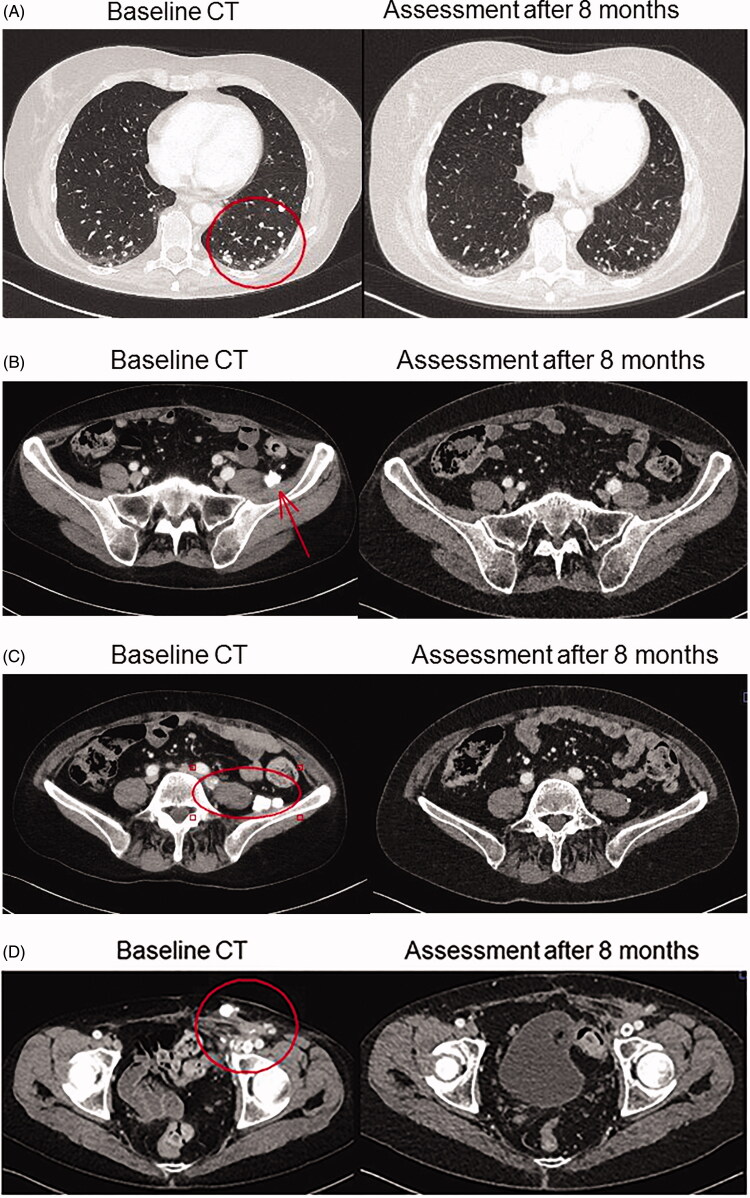
Baseline and eight months CT scans demonstrating complete response for a patient with low-grade serous ovarian cancer treated with combination of BRAF and MEK inhibitors. (A) Lung metastases. (B, C) Lymph node metastases at left pelvic wall. (D) Lymph node metastases in left groin.

In the autumn of 2016, therapy-resistant rapid progression and spread of the disease were evident. Miliary small calcified lung metastases were present with obstructive breathing. Node metastases in the abdomen and the left groin caused pain and lymph-oedema in the left leg. Performance status was fair, ECOG 1–2. However, there was a rapid exponential increase of the serum tumour marker CA-125. Targeted next-generation sequencing (a custom HaloPlex/Agilent capture and Illumina MiSeq sequencing) was performed on a lymph node metastasis, and an activating p.V600E BRAF mutation was detected (16). Combination treatment with the BRAF-inhibitor dabrafenib and the MEK-inhibitor trametinib at full doses was initiated in December 2016, after informed consent from the patient had been obtained. Initially, during the first month, three short treatment interruptions, for 2–3 days each, were necessary to handle repeated fever reactions up to 40 °C (common side effect of dabrafenib), which prompted dose reduction. After one month of treatment, a more than 50% decrease of CA-125 from 3900 to 1668 U/mL was noted. After 35 days, oral prednisolone 10–20 mg daily was introduced to control the fever episodes, as described by Lee at al. (17). This measure was effective and enabled reintroduction of dabrafenib at full dose, while the prednisolone dose could be reduced and kept fairly low. Prompt symptom relief and rapid further decrease of CA-125 levels followed, with normal serum level achieved after 8 months of BRAF–MEK inhibitor combination therapy (Figure 1). A complete response was also verified radiologically after 8 months (Figure 2). After a total treatment period of one year, therapy was stopped in December 2017, as was the prednisolone medication. Six months later, CA-125 started to rise again. After 12 months, progressive disease was evident radiologically in lymph nodes in the left groin and on the left pelvic wall, with escalating pain. Radiotherapy was given to these lymph nodes, which resulted in a partial response and pain relief. Half a year later, in July 2019, after confirming that the tumour still harboured a BRAF p.V600E mutation, combined treatment with BRAF and MEK inhibitors was reintroduced, due to progressive disease with multiple lung metastases. This time, the BRAF inhibitor encorafenib and MEK inhibitor binimetinib were given due to the side effects experienced with dabrafenib and trametinib. After treatment at full doses for five months, the encorafenib dose was reduced, due to side effects, including abdominal pain and bowel discomfort. Since the side effects were successfully treated and a rise in CA-125 was evident along with radiological progression, full doses of encorafenib and binimetinib were reintroduced, and treatment is today ongoing. At present, 3.5 years after initiation of treatment, the response is assessed as stable disease. The patient is in fairly good shape, fully ambulatory with performance status ECOG 1.

Consent for publication in print has been obtained from the patient.

## Discussion

Our patient with metastatic LGSOC had symptomatic and widespread disease, resistant to conventional therapy, rapid progression, and no obvious treatment alternative. However, with next-generation sequencing, a BRAF p.V600E mutation was detected. This finding, the good results of BRAF–MEK combination treatment in malignant melanoma (12), and a recently published case reporting a response on single treatment with a BRAF inhibitor in a patient with LGSOC (10) formed the rationale to initiate BRAF–MEK inhibitor combination therapy for our patient. Furthermore, experiences in melanoma show that with single-drug dabrafenib therapy, the risk for some adverse reactions is higher than with combination treatment with dabrafenib and trametinib (18). Thus, we decided to start combination therapy.

The grading system for malignant serous ovarian carcinoma has changed and is today binary (i.e. low grade or high grade), which has performed better for outcome prediction than the old three-tier grading (1,19). In low-grade serous ovarian carcinoma the mitogen-activated protein kinase (MAPK) pathway is activated, a kinase cascade that mediates the transmission of growth signals into the nucleus, via mutations in KRAS and BRAF, the upstream regulators of the MAPK pathway. Expression of active MAPK has been detected in up to 80% of LGSOC and 78% in serous borderline tumours (4).

Mutations in BRAF, KRAS, and NRAS genes have been reported in LGSOC (3,4,20). In a review of earlier studies the frequency of BRAF mutations ranged from 23% to 48% in serous borderline tumours, and from 0% to 33% in low-grade serous cancers (average 5%) (3).

We report a long-lasting complete response with combined BRAF and MEK inhibition in a patient with advanced LGSOC having received established treatments. The patient is still benefiting from treatment 3.5 years after start of therapy. In comparison, in malignant melanoma patients the median PFS is 11–15 months, and complete response is observed in 8–13% of the cases (14,18). There are a few recent reports on BRAF and MEK inhibitor combination therapy in patients with LGSOC (6,11). However, to our knowledge, there is no previously described case where complete response has been achieved in LGSOC with this treatment and no prior report on a patient benefiting for years.

In conclusion, we demonstrate that complete response and long-term PFS can be achieved in advanced LGSOC with combined BRAF and MEK inhibitor treatment. Hence, this treatment may be an option when established medical treatments, i.e. hormonal and chemotherapy, are no longer effective.
